# Endoplasmic reticulum stress impairment in the spinal dorsal horn of a neuropathic pain model

**DOI:** 10.1038/srep11555

**Published:** 2015-06-25

**Authors:** Enji Zhang, Min-Hee Yi, Nara Shin, Hyunjung Baek, Sena Kim, Eunjee Kim, Kisang Kwon, Sunyeul Lee, Hyun-Woo Kim, Yong Chul Bae, Yonghyun Kim, O.-Yu Kwon, Won Hyung Lee, Dong Woon Kim

**Affiliations:** 1 Department of Anatomy, Brain Research Institute, Chungnam National University School of Medicine, Daejeon 301-747, South Korea; 2 Department of Anesthesia and Pain Medicine, Chungnam National University Hospital, Daejeon 301-721, South Korea; 3 Department of Physiology, Brain Research Institute, Chungnam National University School of Medicine, Daejeon 301-747, South Korea; 4 Department of Oral Anatomy and Neurobiology, School of Dentistry, Kyungpook National University, Daegu 700-412, South Korea; 5 Department of Anesthesiology, Yanbian University Hospital, Yanbian, 133000, China; 6 Department of Chemical and Biological Engineering, The University of Alabama, Tuscaloosa, AL, USA

## Abstract

Endoplasmic reticulum (ER) stress has been implicated in neurodegenerative diseases, but its role in neuropathic pain remains unclear. In this study, we examined the ER stress and the unfolded protein response (UPR) activation in a L5 spinal nerve ligation (SNL)-induced rat neuropathic pain model. SNL-induced neuropathic pain was assessed behaviorally using the CatWalk system, and histologically with microglial activation in the dorsal spinal horn. L5 SNL induced BIP upregulation in the neuron of superficial laminae of dorsal spinal horn. It also increased the level of ATF6 and intracellular localization into the nuclei in the neurons. Moreover, spliced XBP1 was also markedly elevated in the ipsilateral spinal dorsal horn. The PERK-elF2 pathway was activated in astrocytes of the spinal dorsal horn in the SNL model. In addition, electron microscopy revealed the presence of swollen cisternae in the dorsal spinal cord after SNL. Additionally, inhibition of the ATF6 pathway by intrathecal treatment with ATF6 siRNA reduced pain behaviors and BIP expression in the dorsal horn. The results suggest that ER stress might be involved in the induction and maintenance of neuropathic pain. Furthermore, a disturbance in UPR signaling may render the spinal neurons vulnerable to peripheral nerve injury or neuropathic pain stimuli.

Endoplasmic reticulum (ER) stress is caused by disturbances in the structure and function of the ER. Cellular stress, including glucose deprivation, depletion of ER Ca^2+^ stores, exposure to free radicals, and accumulation of unfolded or misfolded proteins, can disrupt proper functioning, leading to the initiation of a cell stress response such as the unfolded protein response (UPR)^1^,^2^,^3^. The UPR is mediated by three ER stress receptors: PKR-like ER kinase (PERK), inositol-requiring enzyme 1 (IRE1), and the activating transcription factor-6 (ATF-6). Indeed, UPR pathways that affect various cell signaling, neuronal connectivity, and cell death processes are a common sign in numerous neurodegenerative disease^2^,^4^,^5^.

ER stress has been suggested to be involved in some neuronal diseases, such as Parkinson’s disease^6^, Alzheimer’s disease^7^, prion disease^8^, and other disorders^9^. However, the exact contributing factors and causes of ER stress in the neuropathic pain processes are not known. In a previous study, we established that autophagy was activated in a spinal nerve ligation (SNL)-induced neuropathic pain model^10^. As a common cytoprotective mechanism, autophagy controls homeostasis in various biological aspects, such as protein and organelle turnover^11^. Moreover, autophagy is associated with the ER at various molecular levels. ER stress can induce autophagy in mammalian cells via several canonical UPR pathways^12^. Therefore, we hypothesized that ER stress may play a critical role in SNL-induced autophagy in the spinal neurons of the dorsal horn. In this study, we examined the expression of three UPR pathway-related genes and proteins to investigate the mechanism underlying SNL-induced ER stress in the spinal dorsal horn.

## Results

### Induction of SNL-induced neuropathic pain in rats

**Figure 1:**
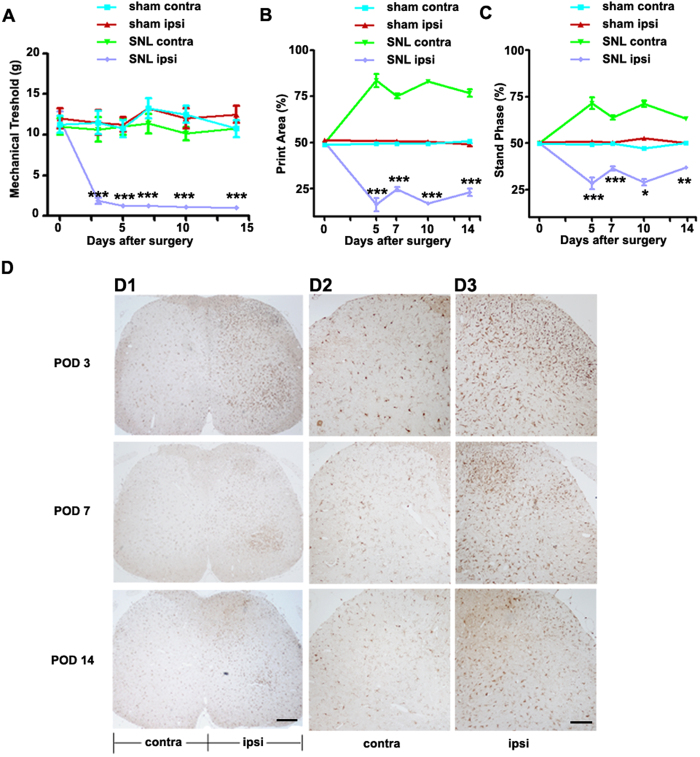
L5 spinal nerve ligation induced mechanical hypersensitivity in the ipsilateral hind paw and microglial activation in the dorsal spinal cord. (**A**) The mechanical threshold was measured on days 0 (baseline), 3, 5, 7, 10, and 14 after surgery. SNL-induced mechanical hypersensitivity was analyzed Von Frey test. ***p < 0.001, *n* = 8. (**B**,**C**) Analysis of Print Area and Stand Phase using the CatWalk system indicated significant differences between the ipsilateral and contralateral paws following SNL. *p < 0.05, **p < 0.01 ***p < 0.001, *n* = 8. (**D**) L5 SNL induced microglial activation in the ipsilateral superficial laminae of the spinal dorsal horn. Spinal cord sections from L5 SNL models were mounted on glass slides and processed for Iba1 immunohistochemistry. The immunoreactivity of microglia was significantly higher in the ipsilateral (D3) superficial laminae of the dorsal horn, when compared with the respective contralateral counterparts (D2). Scale bars = 50 μm in D1, 20 μm in D2–3.

To determine mechanical hypersensitivity following SNL, we measured rat paw withdrawal thresholds (PWTs). SNL-induced mechanical hypersensitivity caused a significant decrease in PWTs on the ipsilateral side (but not on the contralateral side) from day 3 post-surgery (*p* < 0.001) compared with baseline, an effect that persisted up to 14 days (*p* < 0.001, Fig. 1A). In contrast, sham surgery did not cause mechanical hypersensitivity on the ipsilateral or contralateral side.

With the CatWalk system, many static and dynamic gait parameters can be measured. Some of these, including the mean intensity of the paw Print Area and Stand Phase of the hind paw, have been linked to mechanical allodynia and neuropathic pain^13^. In the present study the following individual paw parameters were used: Print Area, which is the surface area of the complete print; and Stance Phase, which is the duration of contact of a paw with the glass plate (in seconds). Print Area is the surface area of the complete print and must be at its minimum as large as the Max Contact Area. A significant decrease in the Print Area and Stand Phase of the ipsilateral paw was observed from post-operative day (POD) 5 (*p* < 0.001) to POD 14 (*p* < 0.001) relative to baseline and the contralateral paw (Fig. 1B).

Because spinal microglia play a key role in neuropathic pain, we examined microglial activation in our SNL model using immunohistochemical analysis of Iba1, a microglial marker. Microglial activation was examined in the spinal cord on days 3, 7 and 14 following L5 SNL. SNL persistently induced the upregulation of Iba1 in the ipsilateral spinal cord, especially in the superficial lumbar dorsal horn, while a few Iba1-immunoreactive (IR) cells could be detected in the contralateral spinal dorsal horn (Fig. 1C).

### Intracellular BIP protein expression in the lumbar dorsal horn after SNL

The molecular chaperone BIP (Grp78) has a range of functions within the ER. It maintains specific transmembrane receptor proteins involved in initiating signaling downstream of the UPR in an inactive state by binding to their luminal domains. BIP recruitment to chaperone misfolded proteins results in BIP dissociation from its conformational binding state to the transmembrane receptor proteins PERK, IRE1, and ATF6^14^. We therefore first focused on the detection of BIP expression.

**Figure 2:**
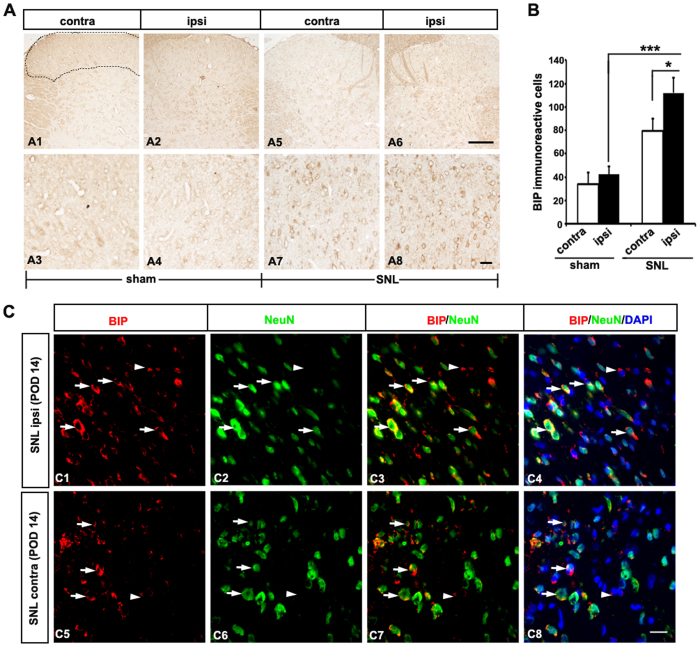
Spinal nerve ligation induced BIP upregulation in ipsilateral dorsal horn neurons. (**A**) Immunochemical staining of BIP was performed on the spinal dorsal horn of the sham group (A1–4) and the SNL group (A5–8) 14 days after the surgery. L5 SNL induced BIP upregulation in the ipsilateral superficial laminae of the dorsal horn (A6,8) relative to the contralateral horn of the lumbar spinal cord (A5, 7). (**B**) The mean numbers of BIP-immunoreactive (IR) cells in the ipsilateral superficial laminae of the dorsal horn were significantly higher than those in the respective contralateral counterparts (*p < 0.05) and the sham group (***p < 0.001). Mean ± SEM, *n* = 6. (**C**) BIP-IR cells in the superficial dorsal horn ipsilateral (C1-C4) and contralateral (C5-C8) were co-labeled primarily with NeuN, a neuronal marker (arrows), although a minority of BIP-IR cells were NeuN-negative (arrowheads). Scale bars = 40 μm in A1, A2, A5, and A6; 20 μm in A3, A4, A7-8, and and **C**.

The expression of BIP was detected by immunohistochemical analysis using specific antibodies in the spinal dorsal horn. L5 SNL induced BIP upregulation in the superficial laminae of the ipsilateral lumbar dorsal horn, compared with the contralateral (*p* < 0.05) and sham group (*p* < 0.001; Fig. 2A,B). L5 SNL also induced BIP upregulation in the superficial laminae of the contralateral lumbar dorsal horn compared with the sham group (*p* < 0.01). To examine the cellular localization of BIP expression, double immunofluorescence staining was performed. BIP IR cells colocalized mostly with NeuN, a neuronal marker, in the superficial dorsal horn (Fig. 2C). These results indicate that ER stress was induced in most neuronal cells of the spinal dorsal horn following L5 SNL.

### Intracellular ATF6 location in the nuclei of neurons of the dorsal horn following SNL

**Figure 3:**
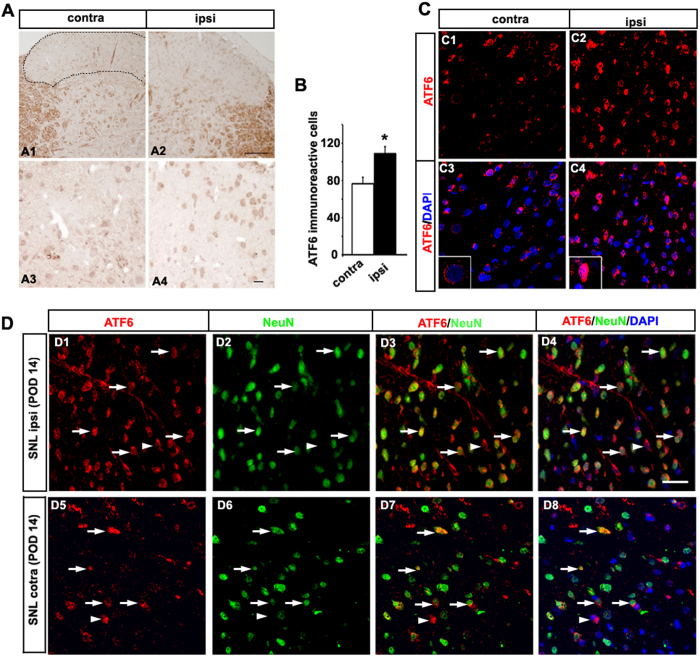
ATF6 is induced in the neuronal nuclei of the ipsilateral dorsal horn on postsurgery day 14. (**A**) L5 SNL induced up-regulation of ATF6 in the ipsilateral lumbar spinal dorsal horn (A2, A4) compared with the contralateral spinal dorsal horn (A1, A3). (**B**) The mean numbers of ATF6-immunoreactive (IR) cells in the ipsilateral superficial laminae of the dorsal horn were significantly higher than those of the respective contralateral counterparts (*p < 0.05). Mean ± SEM, *n* = 6. (**C**) Immunofluorescence staining of ATF6 (red) was performed in the spinal dorsal horn following SNL at post-operative day 14. Nuclei were counterstained with DAPI. L5 SNL induced upregulation of nuclear ATF6 expression in the ipsilateral lumbar spinal dorsal horn (C2, C4) compared with the contralateral spinal dorsal horn (C1, C3). (**D**) The majority of ATF6-immunoreactive (IR) cells were positive for NeuN (green), a neuronal marker of the superficial dorsal horn (arrows), although some ATF6-IR cells were NeuN-negative (arrowheads) in the ipsilateral (D1-D4) and contralateral (D5-D8). Scale bars = 40 μm in A1 and A2; 20 μm in A3–4, **C**, and **D**.

Among UPR sensor pathways involving PERK, IRE1-XBP1, and ATF6, we first examined the translocation of ATF6 using an immunofluorescence assay. Proteolytic activation of ATF6 in the ER stress response is induced by membrane-bound factors, and this process has been termed “regulated intra-membrane proteolysis” (RIP)^15^. The cytoplasmic fragments of ATF6 move to the nucleus and activate the expression of ER stress target genes by binding to the ER stress response element (ERSE) within their promoter regions^16^. Our results showed an upregulation of ATF6 induced by L5 SNL in the ipsilateral lumbar spinal dorsal horn when compared to the contralateral (*p* < 0.05) and the sham groups (*p* < 0.001; Fig. 3A,B). Double immunofluorescence staining was performed to assess the localization of ATF6 IR cells using DAPI, a general nuclear marker, in the superficial dorsal horn. While normally the ATF6 IR area was located in the cytoplasm, the positive fluorescence area was more visible within the nucleus in the ipsilateral spinal dorsal horn when neuropathic pain was induced for 14 days (Fig. 3C). We also found ATF6 IR cells colocalized with NeuN in the superficial dorsal horn on POD14. This suggests that the ATF6 pathway, one of the UPR pathways involved in ER stress, was activated in the SNL dorsal horn neuron.

### Increased BIP mRNA expression and XBP1 splicing in the lumbar dorsal horn after SNL

**Figure 4:**
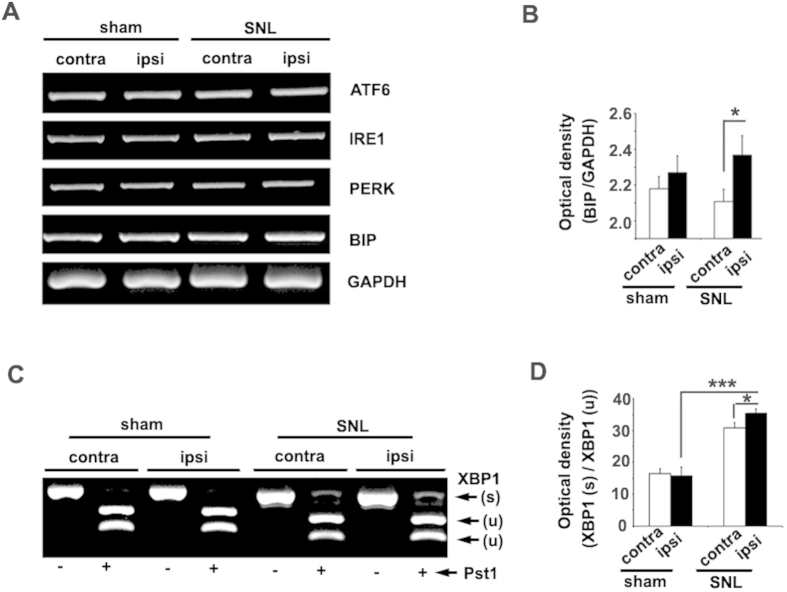
Spinal nerve ligation induced upregulation of BIP and spliced XBP1 mRNA, but not UPR-mediating receptor mRNA, in the spinal dorsal horn on postsurgery day 14. (**A**,**B**) The levels of mRNAs encoding UPR-mediating receptors (ATF6, IRE1, PERK) and BIP were measured in the lumbar dorsal horn by RT-PCR. SNL model animals showed higher BIP mRNA levels than those of sham animals, but normal ATF6, IRE1, and PERK expression. (**C**) The level of spliced XBP1 mRNA was also examined in the SNL model. To distinguish the unspliced and spliced transcripts, whole XBP PCR products were digested with *Pst*I, the restriction site of which is only present in the unspliced form (u). Spliced XBP1 (s), an essential mediator of the UPR, was elevated significantly in the ipsilateral spinal dorsal horn in the SNL model. (**B**,**D**) The band intensities of the UPR-mediated receptor genes BIP and spliced XBP1 were quantified using the ImageJ software, after normalization to GAPDH or unspliced XBP1 (*n* = 3). *p < 0.05, ***p < 0.001.

To study the ER stress response in neuropathic pain quantitatively, we examined UPR gene expression in the lumbar (L4-L6) dorsal horn in both the SNL model and the control group. Intracellular ER stress-related mRNA (ATF6, IRE1, PERK, BIP) expression was assessed by semi-quantitative PCR. We found that BIP mRNA levels, but not ATF6, IRE1, or PERK, were increased significantly on the ipsilateral compared with the contralateral spinal dorsal horn and sham groups (Fig. 4A,B). Quantitative measurements of spliced XBP1 mRNA were used as an indicator of ER stress. Spliced XBP1 mRNA, induced by activated IRE1, is translated into a potent transcription factor that induces BIP expression^17^. To distinguish the unspliced band from the spliced band, PCR products were digested using *Pst*I (which cuts only the unspliced cDNA). The splicing of XBP1 was increased in the ipsilateral dorsal horn after SNL compared with the contralateral (*p* < 0.05) and sham group (*p* < 0.001; Fig. 4C,D). XBP1 splicing was also increased on the contralateral side in SNL compared with the sham group (*p* < 0.05). These data suggest that the IRE1-XBP1 pathway was activated in the SNL dorsal horn.

### Increased phospho-eIF2α expression in the lumbar dorsal horn after SNL

**Figure 5:**
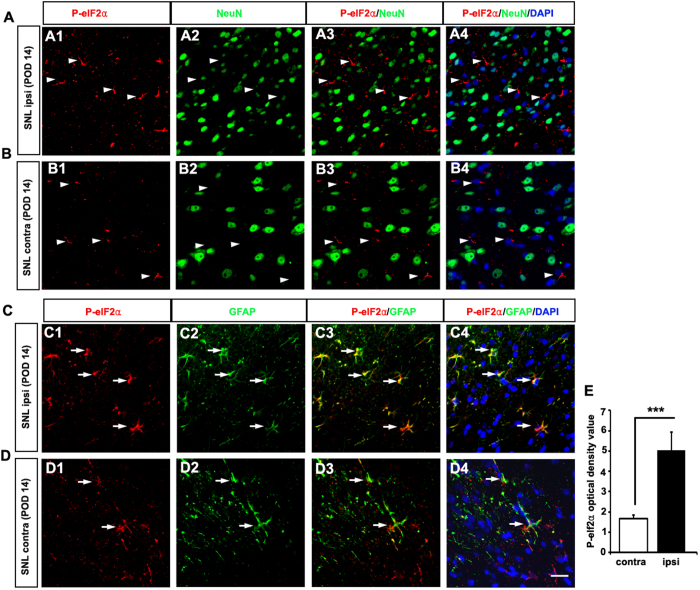
Spinal nerve ligation induced upregulation of phopho-elF2α in astrocytes in the spinal cord on postsurgery day 14. Phospho-elF2α-immunoreactive (IR) cells in the superficial dorsal horn were NeuN-negative (arrowheads in **A**,**B**) in the ipsilateral (**A**) and contralateral (**B**). Some phospho-elF2α IR cells were colabeled with GFAP, an astrocyte marker (arrows in **C**, **D**) in the ipsilateral (**C**) and contralateral (**D**). *p < 0.05, Scale bar = 20 μm.

Three ER stress sensors (PERK, ATF6, and IRE1) implement the UPR. Among them, PERK phosphorylation of the α subunit of eIF2 during ER stress represses protein synthesis, preventing further influx of ER client proteins^18^. Here, we examined phospho-eIF2α expression in the lumbar spinal dorsal horn of the SNL model and the control group. To detect the cellular localization of phospho-elF2α expression, double immunofluorescence staining was performed using antibodies against phospho-elF2α together with NeuN and GFAP, neuronal and astrocytic markers, respectively. The expression of phospho-elF2α was increased in the ipsilateral lumbar spinal dorsal horn, compared with the contralateral (*p* < 0.05; Fig. 5E) and phospho-elF2α IR cells colocalized mostly with GFAP (Fig. 5C,D) in the dorsal horn; however, a few cells colocalized with NeuN (Fig. 5A,B). The results of this double-labeling experiment indicated that the PERK-elF2 pathway was activated in astrocytes in the dorsal spinal cord after SNL.

### Increased ER swelling in dorsal horn neurons after SNL

**Figure 6:**
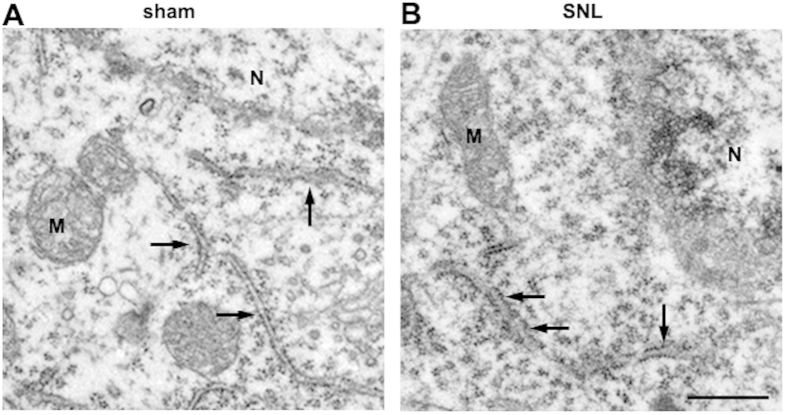
Spinal nerve ligation induced swelling of ER cisternae in neurons of the lumbar dorsal horn on postsurgery day 14. (**A**,**B**) Electron microscopic analysis of neurons in the dorsal spinal cord showed some swollen ER cisternae with medium luminal densities (arrows in **B**), compared with the normally narrow ER cisternae in the ipsilateral spinal dorsal horn of the sham control (arrows in **A**). Scale bar = 500 nm. N: nucleus, M: mitochondria.

Next, we used electron microscopy to evaluate ultrastructural changes in the ER 14 days after SNL. Neurons in the sham spinal cord appeared to be normal, with relatively healthy-looking ER, mitochondria, and nuclei (Fig. 6). Fourteen days after SNL, the mitochondria and the nuclei in the neurons also appeared normal with no appreciable pathological changes; however, several swollen ER lumen were seen, together with mild protein aggregation (Fig. 6). These data suggest that ER stress-induced ER cisternae swelling in the ipsilateral spinal dorsal horn after SNL.

### Inhibition of the ATF6 pathway counteracts SNL-induced neuropathic pain

**Figure 7:**
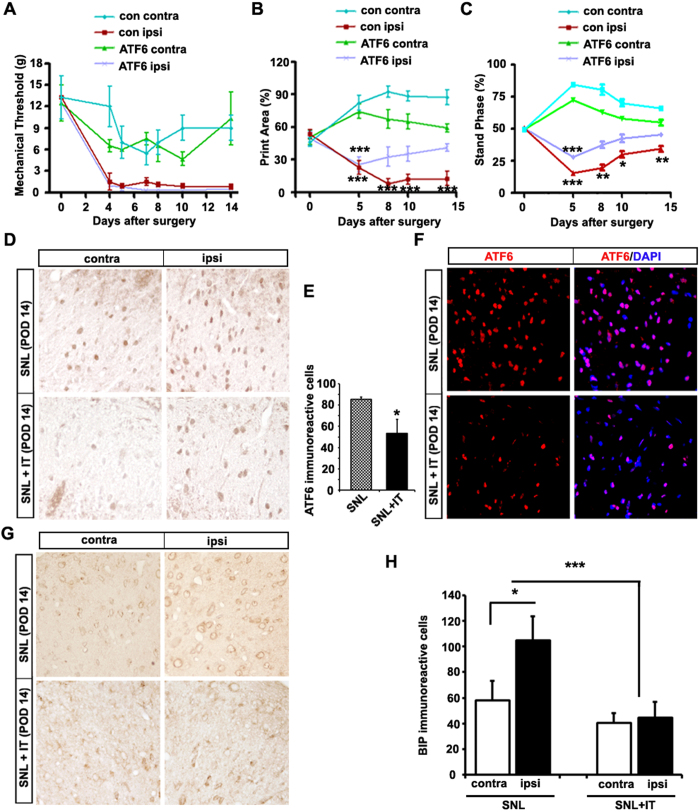
Intrathecal injection of ATF6 siRNA alleviated mechanical hypersensitivity and autophagy. (**A**) The pain behavior of spinal nerve ligation (SNL) animals was assessed from post-operative days (POD) 4 to 14. ATF6 siRNA or vehicle was injected intrathecally on POD 5, 6, and 7. The administration of ATF6 siRNA directly to the spinal cord did not additionally alleviate the mechanical hypersensitivity, as measured using von Frey filaments, compared with the control (vehicle). (**B**,**C**) Intrathecal injection of ATF6 siRNA resulted in significant changes in the Print Area and Stand Phase parameters in the CatWalk system. (**D**–**F**) The expression of ATF6-immunoreactive cells in the dorsal horn after ATF6 siRNA treatment was reduced significantly compared with the contralateral of spinal dorsal horn (*p < 0.05 ). (**G**) Immunohistochemical staining of BIP was performed on sections of the spinal dorsal horn of the SNL group and ATF6 siRNA-treated SNL group at POD 14. ATF6 siRNA treatment reduced BIP expression in the ipsilateral superficial laminae of the spinal dorsal horn compared with the control. Scale bar = 40 μm. (**H**) The mean number of BIP-immunoreactive cells in the dorsal horn after ATF6 siRNA treatment was reduced significantly compared with the control (*p < 0.05, ***p < 0.001). Mean ± SEM, *n* = 6.

To evaluate the behavioral effects of ER stress on neuropathic pain, we examined the loss of function in UPR signaling pathways by transient silencing using ATF6 siRNA. Thus, we examined ATF6 knockdown, because ATF6 signaling is an important marker in response to ER stress, and XBP1 is also induced by activated ATF6^17^. We injected ATF6 siRNA intrathecally on one of three consecutive days (either POD 5, 6, or 7) and then evaluated its effects on POD 8, 10, and 14. The mechanical threshold was measured using Von Frey filaments. However, we found no significant difference in mechanical thresholds after siRNA injection at various time points, compared with the control group (Fig. 7A).

However, CatWalk XT gait analysis indicated that the Print Area and Stand Phase differed significantly according to the ATF6 siRNA injection (Suppl. table 1). The Print Area is basically at least as large as Max Contact Area and shows the ratio of max contact area of contralateral and ipsilateral paw (total percentage of contra- and ipsilateral paw area is 100%). Before SNL, the ratio of contralateral and ipsilateral side was not different. Five days after the surgery, the Print Area of the two ipsilateral paw groups were significantly reduced to 22.60% and 25.58% (both *p* < 0.001 compared to contralateral paw values) at 30 min prior to vehicle and ATF6 siRNA injection, respectively. Print Area in ipsilateral paw recovered from 25.58% to 40.98% compared to contralateral paw in 3, 5, 9 days after ATF6 siRNA injection (Fig. 7B). Before SNL, the duration of the Stance Phase was 50.46% and 50.43% in control and ATF6 siRNA injection groups, respectively. Five days after the surgery, the duration of the Stance Phase of the ipsilateral paw was significantly reduced to 15.56% (*p* < 0.001) and 27.75% (*p* < 0.001) compared to contralateral paw values, respectively, at 30 min before the ATF6 siRNA injection. The Stand Phase was characterized with increased body weight. Therefore we compared the duration time between ispilateral vs. contralateral paw. Stand Phase duration in ipsilateral paw recovered from 40.54% to 26.56% compared to contralateral paw in 3, 5, 9 days after ATF6 siRNA injection (Fig. 7C). This data demonstrated that intrathecal injection of ATF6 siRNA alleviates the mechanical hypersensitivity.

To demonstrate the effects of ATF6 siRNA on the inhibition of UPR pathway, we examined ATF6 expression and BIP expression in the SNL model. When BIP is released from ATF6 during ER stress, site 1 and site 2 proteases cleave ATF6, releasing the cytoplasmic domain into the cytosol. The cleaved domain migrates into the nucleus where it binds to cis-acting ER stress response elements (ERSE) and activates the transcription of ER protein-folding chaperones such as BIP, GRP94, calreticulin, calnexin, and protein disulfide isomerase^19^. Immunochemical staining showed that ATF6 siRNA decreased ATF6 expression and BIP expression in the ipsilateral lumbar spinal dorsal horn, compared with the control group ipsilateral spinal dorsal horn (Fig. 7D–H). Together, these data suggest that inhibition of the ATF6-mediated ER stress response reduced the pain behavior.

## Discussion

Three major ER-resident proteins have been identified as sensors of ER stress: IRE1, PERK, and ATF6. When these stress-sensor proteins are activated by the presence of unfolded proteins in the ER, a signaling cascade is initiated, leading to the regulated expression of a specific subset of stress-response genes^20^. The transcriptional activation of BIP has been used extensively as a standard indicator of UPR initiation, a process that has numerous implications in health and disease^21^,^22^. The activation of IRE1 induces splicing of XBP-1 mRNA via cleavage of its intron. Spliced XBP-1 then functions as a transcription factor for ER stress-related genes^23^. In this study, we found that the BIP (Fig. 2) and Spliced XBP1 (Fig. 4) were interestingly increased in the SNL spinal dorsal horn of contralateral. Cheng *et al*. found that in rats, satellite glia in the contralateral dorsal root ganglion (DRG) are activated by tumor necrosis factor-α (TNF-α) after SNL. TNF-α diffuses from the injured side via cerebrospinal fluid, and activates satellite glia in the contralateral DRG to produce extra nerve growth factor (NGF), which enhances nociceptor excitability, inducing mirror-image pain^24^. Together with our results, these suggest that ER stress might be involved in mirror-image pain.

The activation of PERK results in phosphorylation of the α-subunit of eIF2, leading to translational repression^25^. In addition, the activation of ATF6 is mediated through BIP, which is translocated from the ER to the Golgi complex, after which the cleaved 50-kDa cytoplasmic fragment activates the transcription of UPR target genes^26^,^27^. In this study, we demonstrated that the major chaperone BIP was activated, followed by the IRE-XBP1 and ATF6 pathways but not the PERK-elF2 pathway, in the neurons of the dorsal spinal cord in the SNL model. We showed above that PERK-elF2 pathway, one of the pathways of ER stress, was involved in SNL dorsal horn astrocytes. Therefore, we expect there may be a different function in PERK-elF2 pathway in SNL model compared to IRE-XBP1 and ATF6 pathways.

To investigate the role of ER stress impairment in the development of neuropathic pain in a SNL model, we inhibited ATF6 by injecting siRNA intrathecally. ATF6 siRNA treatment significantly reduced pain behavior, suggesting that an accumulation of ER stress markers during ER stress might be involved in the induction and maintenance of neuropathic pain. Additionally, it suggests that a disturbance in UPR pathway may render neurons vulnerable to peripheral nerve injury or neuropathic pain stimuli. Thus, determination of the mechanisms of ER stress in the spinal cord during neuropathic pain may provide beneficial information to guide the development of novel pharmacological treatments.

In this study, we demonstrated that UPR pathway was activated in the SNL model. We hypothesized that ER stress play an important role in the neuropathic pain maintenance. This is now supported by the following findings. First, using an SNL model, we demonstrated that UPR pathway markers were increased from SNL spinal dorsal horn in a response. Second, we detected several swollen ER lumen in the SNL model of the spinal cord dorsal horn using electron microscopy. Third, we showed that SNL-induced pain behavior was diminished by inhibition of ATF6, an indicator of ER stress. Unfolded or misfolded proteins in the ER lumen are retrotranslocated through the translocon to the cytoplasm, where they are usually ubiquitinated and degraded in the proteasome^28^. Similarly, the upregulation of autophagy-related genes (LC3, Gabarapl1, Bnip3, Atg4b, and Atg12l) has been documented at the transcriptional and translational levels in several other species under ER stress^29^,^30^,^31^.

During autophagy, cytoplasmic constituents (including misfolded or aggregated proteins), damaged organelles (such as mitochondria, ER components, and peroxisomes), and intracellular pathogens are sequestered into double-membrane autophagosomes, where their contents are degraded by lysosomal hydrolases^32^. Ogata *et al*. reported that unfolded proteins were processed by both the “ER-associated protein degradation” (ERAD) and autophagy systems, and disturbance of these systems resulted in ER stress and cell damage^12^. More importantly, autophagy is involved in the maintenance of ATP production via the catabolism of intracellular substrates, representing a mechanism of cell survival after growth factor withdrawal^33^. Thus, it also seems conceivable that autophagy caused by ER stress allows the maintenance of energy homeostasis to prevent cell death.

Recently, it was reported that autophagic imbalance can impact basal cell functions, which has been implicated in neuropathic pain^34^,^35^. In a previous study, we established that autophagy was activated in a SNL-induced neuropathic pain model^10^. As a common cytoprotective mechanism, autophagy controls homeostasis in various biological aspects, such as protein and organelle turnover^11^. Moreover, autophagy is associated with the ER at various molecular levels. ER stress can induce autophagy in mammalian cells via several canonical UPR pathways^12^. Although the connection between ER stress and autophagic activity at the molecular level has been established, the interplay between the two phenomena, especially in the spinal cord during neuropathic pain, requires further research.

## Materials and Methods

### Animals and surgical procedure

Male Sprague-Dawley rats (180–200 g, Koatech, Korea) were housed individually in cages on a standard 12:12 h light:dark cycle. Water and food were provided to the rats *ad libitum*. Rats were transported to the laboratory approximately 1 h prior to each experiment. All experiments were carried out with the approval of the Animal Care and Use Committee at the Chungnam National University (CNU-00491) and were accordance with the ethical guidelines of the National Institutes of Health and the International Association for the Study of Pain. The same method and procedure for surgery were used as a previously published paper^10^.

### Pain threshold assessment

#### Withdrawal threshold

Mechanical paw withdrawal thresholds (PWTs) were measured using the up-down Von Frey testing following spinal nerve ligation or sham surgery. The same methods and procedure were used as a previously published paper^10^.

CatWalk gait analysis was done on day 5, 7, 10 and 14. The animals traverse a walkway with a glass floor located in a darkened room. In general, rats cross the CatWalk runway easily and at a constant speed. The CatWalk analysis system consists of a glass walkway which contains light from a white fluorescent source. The light rays from this source exhibit the complete internal reflection. When an object touches the glass runway, the light is reflected downwards, where it is detected by a video camera. This signal is then digitized, which allows analysis by the CatWalk program software (CatWalk XT version 10.5.505, Nodus).

### RNA isolation and Reverse transcription polymerase chain reaction (RT-PCR)

Total RNA was isolated from the ipsilateral and contralateral dorsal horn of rat lumbar using Trizol Reagent (Ambion). cDNA was synthesized using 1 g of total RNA and QuantiTect Reverse Transcription Kit (Qiagen). cDNA was amplified by PCR using specific primer (Suppl. Table S2). Reaction products were analyzed on 1–2% agarose gels, and the bands were visualized by ImageJ software.

### Immunohistochemistry and double immunofluorescence

Immunochemistry and immunofluorescence experiments were performed as previously described for lumbar enlargement (L4–L6) regions of the spinal cords^10^, excepting the antibodies. Here, we used polyclonal antisera against BIP antibody (1:2000, Abcam, #ab21685) or ATF-6α (1:500, H-280, Santa Cruz, #sc-2799).

### Electron microscopic examination

Representative photomicrographs of samples were taken from three rats in each group following SNL or sham surgery. After 14 days from the surgery, the rats were perfused with pre-cooled phosphate buffered saline solution (PBS, pH 7.4) followed by 2.5% glutaldehyde, 1% PFA, 0.1% Picric acid in 0.1 M PB after anesthetization. The lumbar enlargement (L4–L6) regions of the spinal cords were removed and kept in 2.5% glutaraldehyde in 0.1 M PBS (pH 7.4). Ipsilateral dorsal horn of rat was selected for analysis. The selected areas were processed by post fixation in 1% osmium tetroxide for 1 h, dehydrated in graded ethanol and embedded in epoxy resin. Polymerization was performed at 80 °C for 24 h. Blocks were cut on a Reichert ultramicrotome into ultrathin sections (60 mm), which were post-stained with uranyl acetate and lead citrate, and viewed under a Hitachi 7100 electron microscopy.

### RNAi administration

Neuropathic pain was induced in rats by SNL surgery. The rats were divided into siRNA (Rn-Atf6-predicted-1, SI04729928, Qiagen) and vehicle groups, with at least six rats per group. siRNA and vehicle doses were prepared immediately just before administrating the RNA solution (200 μM in annealing buffer) with a transfection reagent, (Invitrogen, #11668-019), at a 1:4 ratio (w:v). At this ratio, the final concentration of RNA as an RNA/lipid complex was 2 μg in 10 μL. Then, 10 μL of siRNA or Lipofectamine with scrambled siRNA (defined as vehicle) were delivered to the lumbar region of the spinal cord via intrathecal (IT) injection. Injections were given daily for three consecutive days. Nociceptive testing and tissue collection were carried out at days after SNL.

### Statistical analysis

Results from the behavioral study was used two-way ANOVA, followed by *Tukey post boc* test. The results of immunoblot analysis and immunohistochemical analyses were analyzed statistically by one way ANOVA or student’s t-test. All data are presented as means ± SEM. Significance was set at *p < 0.05, **p < 0.01, ***p < 0.001. The statistical software package SigmaStat (Systat, San Jose, CA) was used to perform all statistical analyses.

## Additional Information

**How to cite this article**: Zhang, E. *et al*. Endoplasmic reticulum stress impairment in the spinal dorsal horn of a neuropathic pain model. *Sci. Rep*. **5**, 11555; doi: 10.1038/srep11555 (2015).

## Supplementary Material

Supplementary Information
