# Supplementary material for: Endoplasmic reticulum stress impairment in the spinal dorsal horn of a neuropathic pain model
Source: Sci Rep. 2015 Jun 25;5:11555. doi: 10.1038/srep11555 (PMC4479804; doi:10.1038/srep11555)
Supplement: Supplementary Information [file srep11555-s1.doc]

**Endoplasmic reticulum stress impairment in the spinal dorsal horn of a neuropathic pain model**

Enji Zhang^2,5^, Min-Hee Yi^2^, Nara Shin^2^, HyunJung Baek^2^, SeNa Kim^2^, EunJee Kim^2^, Kisang Kwon^2^, Sunyeul Lee^1^, Hyun-Woo Kim^3^, Yong Chul Bae^4^, O-Yu Kwon^2^, Young Ho Lee^2^, Won Hyung Lee^1*^, Dong Woon Kim^2, *^

**SUPPLEMENTARY INFORMATION**

**Table S1: CatWalk analysis data**

**
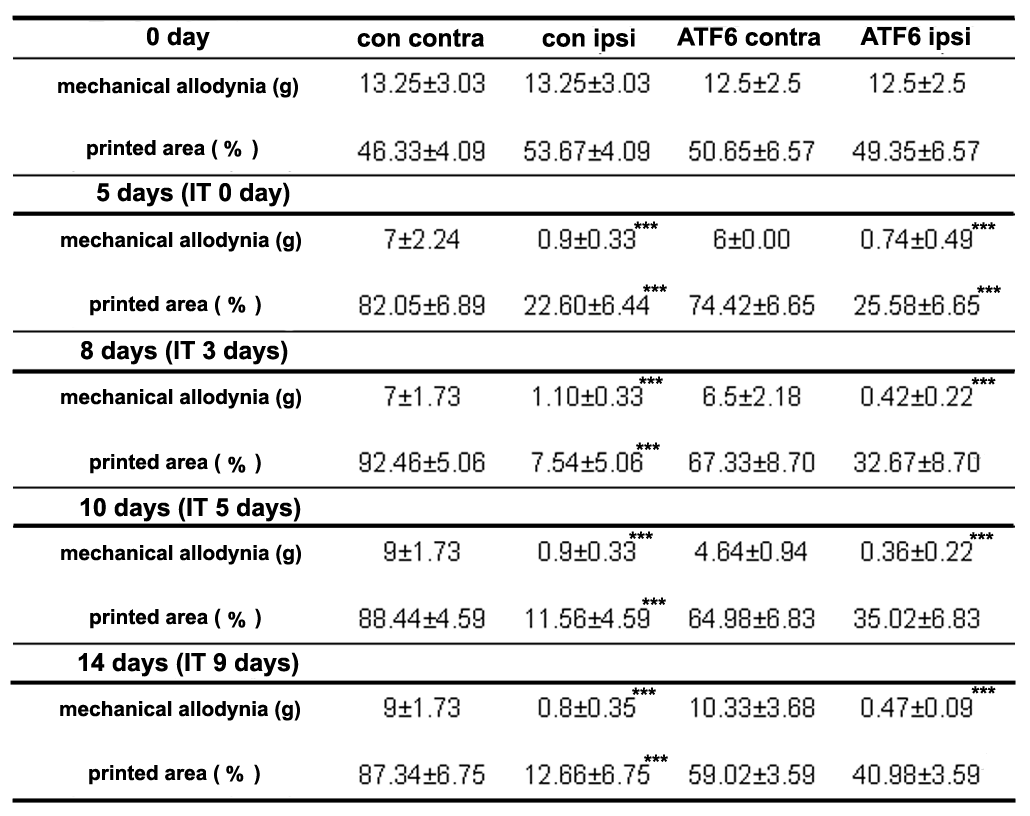
**

**Table S2: List of primers used**

| PCR | **Sequence (5’ > 3’)** | **Purpose** |
| --- | --- | --- |
| ATF6 | F: TCA GCT GAT GGC TGT CCA GT  R: TGA TGT GGA GGA TCC TGG TG | PCR of *ATF6* |
| IRE1 | F: CCT CGT GCT TGT CTG AGT GG  R: CCC ATC ACC TTG TT CAT CA | PCR of *IRE1* |
| PERK | F: CCG AAG CCA CCT TGT CTA CC  R: TTC ATC TG GTC CAT TGC AG | PCR of *PERK* |
| BIP | F: TTG GGG ACC ACC TAT TCC TG  R: AAC TGC ATG GGT GAC CT | PCR of *BIP* |
| XBP1 | F: AAA CAG AGT AGC AGC TCA GAC TGC  R: TCC TCC TGG GTA GAC CTC TGG GAG | PCR of *XBP1* |
| GAPDH | F: ACA TCA AAT GGG GTG ATG CT  R: AGG AGA CAA CCT GGT CCT CA | PCR of GAPDH |


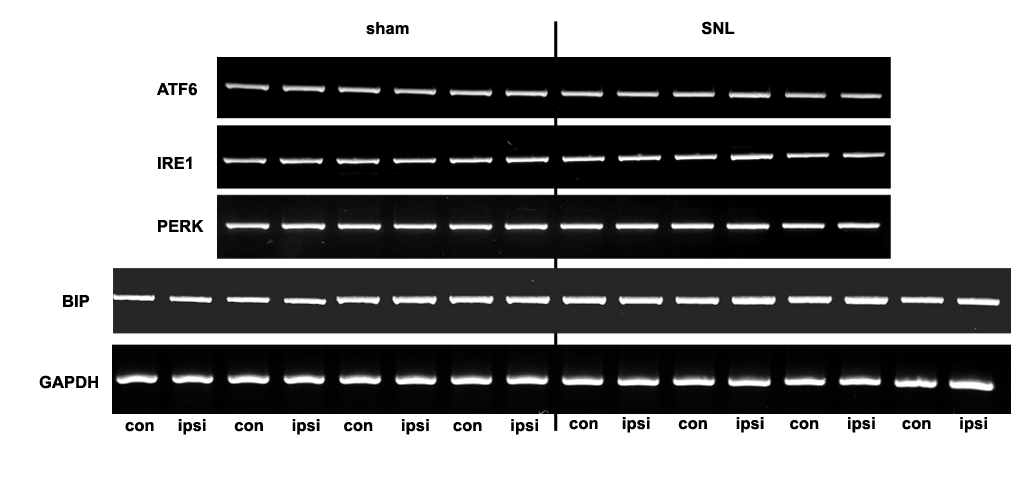


Full length gels of Fig. 4A


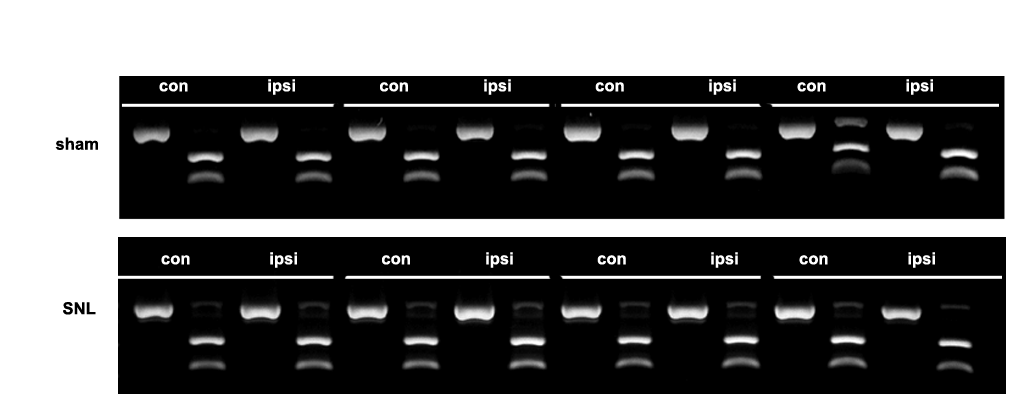


Full length gels of Fig. 4C
